# HDL Cholesterol and Atherogenic Lipid Indices in Hidradenitis Suppurativa: Associations with BMI and Hurley Disease Severity

**DOI:** 10.3390/metabo16100737

**Published:** 2026-09-30

**Authors:** İlkay Özer, Aslıhan Çiçekli, Kübra Karataş, Selami Aykut Temiz, Munise Daye, Recep Dursun

**Affiliations:** Department of Dermatology and Venereology, Faculty of Medicine, Necmettin Erbakan University, 42090 Konya, Türkiye; aslihan.tumkaya@erbakan.edu.tr (A.Ç.); kubra.karatas@erbakan.edu.tr (K.K.); satemiz@erbakan.edu.tr (S.A.T.); mdaye@erbakan.edu.tr (M.D.); rdursun@erbakan.edu.tr (R.D.)

**Keywords:** hidradenitis suppurativa, dyslipidemia, atherogenic index of plasma, Castelli risk indices, atherogenic coefficient, cardiovascular risk, Hurley stage

## Abstract

**Background:** Hidradenitis suppurativa (HS) is a chronic inflammatory disease associated with cardiometabolic comorbidities and increased cardiovascular risk. **Methods:** This retrospective case–control study evaluated serum lipid parameters and atherogenic indices—including the atherogenic index of plasma (AIP), Castelli risk index I (CRI-I), Castelli risk index II (CRI-II), and the atherogenic coefficient (AC)—in 176 patients with HS compared with 132 controls frequency-matched for age and sex and examined their associations with BMI and Hurley stage using multiple linear regression models. **Results:** BMI was significantly higher in patients with HS than in controls (median 29.4 vs. 26.1 kg/m^2^, *p* < 0.001), and subsequent regression analyses were therefore adjusted for BMI. Patients with HS exhibited higher triglyceride, LDL-C, and non-HDL-C levels, lower HDL-C levels, and higher atherogenic indices than controls, whereas total cholesterol did not differ significantly between the groups. After BMI adjustment, differences in triglyceride, LDL-C, and non-HDL-C lost significance, whereas HDL-C and all atherogenic indices remained significantly altered. In ordinal trend analyses, increasing Hurley stage showed modest associations with lower HDL-C and higher LDL-C, non-HDL-C, CRI-I, CRI-II, and AC, whereas triglycerides and AIP showed no significant trends after FDR correction. **Conclusion:** However, residual confounding by unmeasured cardiometabolic and lifestyle factors cannot be excluded, and the present findings do not establish increased cardiovascular risk or the clinical predictive utility of these indices. These findings should therefore be considered hypothesis-generating and warrant confirmation in prospective studies incorporating comprehensive cardiometabolic covariates, inflammatory biomarkers, and direct measures of subclinical atherosclerosis.

## 1. Introduction

Hidradenitis suppurativa (HS) is a chronic, recurrent inflammatory skin disease characterized by painful nodules, papules, pustules, abscesses, sinus tract formation, and scarring predominantly affecting intertriginous regions, including the axillae, inguinal folds, perineal and perianal areas, and inframammary and intermammary folds [1]. Owing to its chronic course, recurrent flares, and associated physical and psychological burden, HS exerts a profound negative impact on patients’ quality of life [2]. The frequent coexistence of metabolic syndrome, type 2 diabetes mellitus, nonalcoholic fatty liver disease, polycystic ovary syndrome, inflammatory bowel disease, depression, anxiety, dyslipidemia, and atherosclerosis has led to the growing recognition of HS as a systemic inflammatory disorder rather than a disease confined to the skin [3]. A Finnish registry-based study reported substantially reduced life expectancy among individuals with HS compared with the general population; however, this estimate was derived from a specific national population and may not be generalizable to other settings. Excess mortality in patients with HS is driven predominantly by circulatory disorders, particularly cardiovascular diseases, which constitute the leading cause of death in this population, while malignancies, ischemic heart disease, and cerebrovascular diseases also contribute significantly to mortality risk [4].

Dyslipidemia is a common metabolic comorbidity in patients with HS and is considered a key contributor to their unfavorable atherogenic profile. The lipid profile observed in HS is often characterized by reduced high-density lipoprotein cholesterol (HDL-C) levels and elevated triglyceride (TG) concentrations, a pattern consistent with atherogenic dyslipidemia [5]. Emerging evidence suggests that patients with HS may be at an even greater risk of developing atherosclerotic cardiovascular disease than those with other chronic inflammatory dermatoses, including psoriasis and atopic dermatitis [6]. These findings underscore the importance of early cardiovascular risk assessment and appropriate risk management strategies in this patient population.

In recent years, several atherogenic indices derived from lipid ratios, including the atherogenic index of plasma (AIP), Castelli risk indices I and II (CRI-I and CRI-II), and the atherogenic coefficient (AC), have emerged as potentially useful markers of cardiometabolic risk. Accumulating evidence suggests that these composite indices may provide superior predictive performance for cardiovascular events and atherosclerotic burden compared with individual lipid parameters alone. Moreover, they have been increasingly utilized to evaluate cardiovascular and atherosclerotic risk in chronic inflammatory disorders, including inflammatory dermatologic diseases [7,8].

Given the increased cardiovascular morbidity and mortality associated with hidradenitis suppurativa, the present study aimed to evaluate serum lipid parameters and atherogenic indices, including AIP, CRI-I, CRI-II, and AC, in patients with HS and to investigate their associations with BMI and disease severity in this population. We hypothesized that atherogenic lipid indices would remain altered in patients with HS after accounting for BMI and that these abnormalities would be more pronounced in patients with advanced disease.

## 2. Materials and Methods

This retrospective case–control study was conducted following approval by the Ethics Committee of Necmettin Erbakan University (approval no. 2026/6670). Consecutive eligible records of patients diagnosed with HS who attended the dermatology outpatient clinic between January 2024, and May 2026 were included. The control group consisted of age- and sex-frequency-matched patients attending the dermatology outpatient clinic who did not have hidradenitis suppurativa or a systemic inflammatory dermatosis. Eligible controls had non-systemic inflammatory or non-inflammatory dermatologic conditions; examples included tinea corporis, onychomycosis, and melanocytic nevi. These diagnoses are provided as illustrative examples and did not constitute predefined diagnostic categories for control-group selection. Individual control diagnoses were not systematically coded as a study variable in the retrospective dataset. Controls were frequency-matched to patients with HS according to age and sex. Patients receiving medications known to affect lipid metabolism, including lipid-lowering agents and isotretinoin, as well as pregnant or breastfeeding women, were excluded from the study. Medical records of both patients with HS and control subjects were retrospectively reviewed. Demographic characteristics, body mass index (BMI), and fasting serum lipid parameters were recorded.

The following formulas were used to calculate atherogenic indices: Non-HDL-C (Total Cholesterol mg/dL − HDL-C mg/dL), CRI-I (Total Cholesterol mg/dL/HDL-C mg/dL), CRI-II (LDL-C mg/dL/HDL-C mg/dL), AC (Non-HDL-C mg/dL/HDL-C mg/dL), and AIP (log_10_[Triglycerides (mmol/L)/HDL-C(mmol/L)]) were calculated for each participant. For calculation of the atherogenic index of plasma (AIP), triglyceride and HDL-C concentrations measured in mg/dL were first converted to mmol/L using the corresponding conversion factors (triglycerides: mg/dL ÷ 88.57; HDL-C: mg/dL ÷ 38.67). AIP was then calculated as log_10_[TG (mmol/L)/HDL-C (mmol/L)].

Statistical analyses were performed using Python 3.12 (SciPy, NumPy). Continuous variables were assessed for normality using the Shapiro–Wilk test. Welch’s *t*-test was used for normally distributed variables irrespective of a preliminary test of variance equality; therefore, Levene’s test was not used as a decision rule for test selection. Non-normally distributed continuous variables were compared using the Mann–Whitney U test. Because BMI differed significantly between groups, its potential confounding effect was evaluated using multiple linear regression models including study group and BMI as predictors. BMI-adjusted means, regression coefficients, 95% confidence intervals, and adjusted *p*-values were reported. Model assumptions were evaluated by inspection of residual distributions, homoscedasticity, and influential observations. To address multiplicity across the nine lipid parameters and lipid-derived indices evaluated in the HS-versus-control analyses, the Benjamini–Hochberg false discovery rate (FDR) procedure was applied as an additional analysis. As sensitivity analyses, potential effect modification by BMI was examined by adding a group × BMI interaction term to each BMI-adjusted model. Potential non-linearity in the association between BMI and each outcome was additionally evaluated by including a quadratic BMI term (BMI^2^), with BMI centered at its sample mean before construction of the quadratic term. FDR correction was also applied separately across the nine group × BMI interaction tests and the nine quadratic BMI tests. For descriptive assessment of between-group effect magnitude, unadjusted standardized effect sizes were estimated using Hedges’ g based on the observed group data, with positive values indicating higher values in the HS group. These effect-size estimates were descriptive and were not adjusted for BMI. Because Hurley stage is an ordinal measure of structural disease severity, an additional trend analysis was performed with stages I, II, and III. Monotonic associations with increasing Hurley stage were evaluated using Spearman rank correlation. Benjamini–Hochberg FDR correction was applied across the nine lipid parameters and lipid-derived indices in these ordinal trend analyses; BMI was not included in this FDR family. All tests were two-tailed, and *p* < 0.05 was considered statistically significant.

## 3. Results

A total of 308 participants were enrolled in the study, including 176 patients with HS and 132 controls. According to the Hurley staging, 41 (23.3%) patients were classified as stage I, 63 (35.8%) as stage II, and 72 (40.9%) as stage III. No significant differences were observed between the HS and control groups regarding age or sex distribution. While height was comparable between groups, patients with HS exhibited significantly greater body weight and BMI than controls. Detailed demographic and clinical characteristics of the study population are presented in Table 1.

While total cholesterol did not differ significantly between the groups, patients with HS demonstrated significantly lower HDL-C levels and higher triglyceride and LDL-C concentrations than controls. Moreover, non-HDL-C, AC, CRI-I, CRI-II, and AIP values were all significantly higher in the HS group (Table 2).

Body mass index (BMI) differed significantly between the HS and control groups (see Table 1). The extent to which this difference affected lipid/atherogenic index comparisons was evaluated. After adjustment for BMI, the differences in triglycerides, LDL-C, and non-HDL-C were attenuated and were no longer statistically significant. In contrast, HDL-C and all evaluated atherogenic indices (CRI-I, CRI-II, AC, and AIP) remained significantly different between groups after BMI adjustment (Table 3).

Sensitivity analyses provided no FDR-corrected evidence of effect modification by BMI or non-linearity in the associations between BMI and the evaluated lipid outcomes. A nominal group × BMI interaction was observed for AIP (*p* = 0.018), but this did not remain significant after FDR correction (q = 0.159). Similarly, the quadratic BMI term was nominally significant for AIP (*p* = 0.040) but not after FDR correction (q = 0.253). No other group × BMI interaction or quadratic BMI term reached nominal statistical significance.

Significant overall differences across Hurley stages were observed for HDL-C, LDL-C, non-HDL-C, CRI-I, CRI-II, and AC. In Bonferroni-corrected pairwise comparisons, significant differences were predominantly observed between Hurley stages I and III for HDL-C, LDL-C, CRI-I, CRI-II, and AC.

In the additional ordinal trend analysis, increasing Hurley stage was associated with lower HDL-C (Spearman ρ = −0.174, *p* = 0.021, q = 0.031) and higher LDL-C (ρ = 0.201, *p* = 0.007, q = 0.017), non-HDL-C (ρ = 0.186, *p* = 0.014, q = 0.024), CRI-I (ρ = 0.226, *p* = 0.003, q = 0.008), CRI-II (ρ = 0.262, *p* < 0.001, q = 0.004), and AC (ρ = 0.226, *p* = 0.003, q = 0.008). No significant ordinal trends were observed for total cholesterol, triglycerides, or AIP after FDR correction. BMI showed no association with increasing Hurley stage (ρ = −0.006, *p* = 0.941). Overall, the significant ordinal associations were modest in magnitude (Table 4).

## 4. Discussion

In our study, patients with HS exhibited elevated triglyceride, LDL-C, and non-HDL-C levels, along with reduced HDL-C levels; all atherogenic indices (CRI-I, CRI-II, AC, and AIP) were found to be significantly higher in the HS group. Although the differences in triglyceride, LDL-C, and non-HDL-C levels lost statistical significance in BMI-adjusted analyses, HDL-C and all atherogenic indices remained significantly different between groups after adjustment for BMI. Furthermore, patients with more advanced Hurley stages tended to exhibit lower HDL-C and higher LDL-C, CRI-I, CRI-II, and AC values, with significant differences observed particularly between stages I and III. These differences were observed despite the absence of a significant difference in BMI across Hurley stages.

HS, which is currently regarded as a systemic disease, is frequently accompanied by hyperlipidemia, obesity, and metabolic syndrome, and consequently, the risk of cardiovascular disease (CVD), such as myocardial infarction and heart failure, nearly doubles [9]. Elevated triglyceride and reduced HDL-C levels are among the lipid abnormalities reported in HS. According to a comprehensive meta-analysis examining lipid levels in patients with HS, encompassing thirteen studies and 282,871 participants, although total cholesterol and LDL-C levels did not differ from control groups, patients exhibited elevated triglyceride and reduced HDL-cholesterol levels. Accordingly, it has been recommended that lipid profile assessment be performed in patients with HS, and that high-risk patients be evaluated early through a multidisciplinary approach involving cardiologists [10].

In our study, the initially observed differences in triglycerides, LDL-C, and non-HDL-C were attenuated and were no longer statistically significant after adjustment for BMI. This finding suggests that differences in BMI may contribute to the observed between-group variation in these lipid parameters, although the present analysis cannot establish that BMI fully explains these differences. In contrast, lower HDL-C remained significantly associated with HS after adjustment for BMI. Importantly, BMI adjustment alone cannot account for other potentially relevant cardiometabolic and lifestyle factors, including smoking, diabetes, hypertension, insulin resistance, dietary habits, physical activity, alcohol consumption, and systemic treatments. Therefore, the persistence of the HDL-C association after BMI adjustment should not be interpreted as evidence that it is independent of adiposity or other cardiometabolic characteristics. Rather, our findings indicate that the between-group difference in HDL-C was not fully accounted for by BMI in the present analysis. Disease-related inflammatory or metabolic alterations may represent one possible explanation, but this hypothesis requires confirmation in studies incorporating relevant confounders and direct inflammatory measurements.

There are limited studies examining the atherogenic indices derived from lipid profiles—namely CRI I-II, AIP, and AC—in patients with HS. In a study comparing 78 patients with HS to 62 controls, AIP and total cholesterol were found to be significantly higher and HDL-C significantly lower, whereas CRI I-II and AC did not differ significantly [11]. In contrast to the existing literature, we found that all atherogenic indices remained significantly elevated in patients with HS after adjustment for BMI. After adjustment for BMI, HDL-C and the lipid-derived indices remained significantly different between groups. However, because these indices share lipid components—particularly HDL-C—and because residual confounding by unmeasured cardiometabolic factors cannot be excluded, these findings should be interpreted as overlapping features of the observed lipid phenotype rather than as evidence of an HS-specific metabolic or inflammatory mechanism.

Our study also evaluated the relationship between disease severity and lipid values as well as atherogenic indices. Studies comparing disease severity with lipid values in the literature have yielded conflicting results. In a study of thirty patients, hypertriglyceridemia was detected in 43.3% of patients, hypercholesterolemia in 20%, and elevated LDL in 13.3%, while only 16.67% of patients met the NCEP ATP III metabolic syndrome criteria. A significant association was found between disease severity and fasting serum insulin, ESR, and total cholesterol, whereas TG and HDL-C did not show a statistically significant difference across Hurley stages [12]. In a cohort study of 197 patients, no significant associations were observed between Hurley stage and either blood glucose levels or lipid parameters; the authors interpreted these findings as suggesting that dyslipidemia may not be a direct determinant of HS severity but may instead reflect accompanying metabolic and systemic comorbidities [13].

However, in a study evaluating 109 patients with HS and 109 dermatology outpatient controls, increased systemic inflammation was found to be associated with lower HDL levels. In addition, the prevalence of low HDL was observed to be higher in patients with Hurley stage III, although this difference did not reach statistical significance. Nevertheless, the authors suggested that their findings support a possible relationship between increasing disease severity and decreasing HDL levels, attributing the lack of statistical significance to the categorical nature of the Hurley staging system, which may not adequately capture the variability in disease severity, as well as to the limited sample size reducing statistical power [14].

In the present study, several lipid measures were associated with increasing Hurley stage. The original stage-based comparisons showed significant differences in HDL-C, LDL-C, CRI-I, CRI-II, and AC, with post-hoc differences occurring predominantly between Hurley stages I and III. In additional ordinal analysis, increasing Hurley stage showed modest inverse association with HDL-C and positive associations with LDL-C, non-HDL-C, CRI-I, CRI-II, and AC; these associations remained statistically significant after FDR correction. In contrast, triglycerides and AIP showed no significant ordinal trend.

These findings support an association between selected components of the lipid phenotype and greater structural disease severity but should not be interpreted as demonstrating a progressive relationship between dyslipidemia and systemic inflammatory activity. Hurley staging primarily reflects cumulative structural disease burden, including scarring and tunnel formation, and does not directly quantify current inflammatory activity. Furthermore, inflammatory biomarkers and dynamic HS activity measures were not available in the present retrospective cohort. Therefore, the observed associations cannot establish inflammation as the mechanism linking HS severity to alterations in lipid metabolism.

Systemic inflammation represents one biologically plausible mechanism that may contribute to altered lipoprotein metabolism in HS. Previous studies have reported increased inflammatory mediators, including IL-17, TNF-α, IL-1β, and CRP, in HS and have suggested that chronic inflammation may adversely affect lipoprotein metabolism [15]. In addition, previous evidence of impaired HDL functionality provides a potential biological framework for the reduced HDL-C observed in our cohort. A prospective study of 44 patients with HS demonstrated reduced HDL-associated paraoxonase-1 (PON1) activity and increased HDL oxidative susceptibility, with these alterations being associated with disease severity [16]. However, neither inflammatory mediators nor HDL functional measures were assessed in the present cohort. Accordingly, these mechanisms should be considered biologically plausible hypotheses supported by external literature rather than mechanisms demonstrated by our findings.

## 5. Conclusions

Patients with HS exhibited lower HDL-C levels and higher HDL-C-derived atherogenic indices than controls, and these differences persisted after statistical adjustment for BMI and correction for multiple testing. Within the HS group, increasing Hurley stage showed modest associations with lower HDL-C and higher LDL-C, non-HDL-C, CRI-I, CRI-II, and AC. Because the evaluated indices are mathematically interdependent, these findings should be interpreted as complementary features of an overlapping lipid phenotype rather than as independent biomarkers.

The observed associations were not fully accounted for by BMI in the present analysis; however, residual confounding by unmeasured cardiometabolic and lifestyle factors cannot be excluded. Moreover, the study did not assess cardiovascular events, direct measures of subclinical atherosclerosis, inflammatory biomarkers, or HDL functionality. Consequently, these findings do not establish increased cardiovascular risk or the clinical predictive utility of the evaluated indices and should be regarded as hypothesis-generating. Prospective studies incorporating comprehensive cardiometabolic covariates, dynamic measures of HS activity, inflammatory biomarkers, HDL functional assessments, and direct measures of subclinical atherosclerosis are needed to determine the clinical and prognostic significance of this lipid phenotype.

Several limitations should be considered. First, the retrospective, single-center design limits causal inference and generalizability. Although analyses were adjusted for BMI, information on several potentially important cardiometabolic and lifestyle confounders, including smoking, diabetes, hypertension, insulin resistance/metabolic syndrome, dietary habits, physical activity, alcohol consumption, and systemic treatments, was not consistently available. Residual confounding therefore cannot be excluded. Second, Hurley staging primarily reflects cumulative structural disease burden rather than current inflammatory activity, and dynamic HS activity measures and inflammatory biomarkers were not available. Third, the evaluated lipid-derived indices are mathematically interdependent and should not be considered independent biological measures; notably, CRI-I and AC are deterministically related (CRI-I = AC + 1). Finally, the study did not assess cardiovascular events, direct measures of subclinical atherosclerosis or vascular function, or HDL functional properties. Thus, the observed lipid associations cannot establish cardiovascular risk prediction, clinical utility, or underlying inflammatory mechanisms. In addition, controls were selected from dermatology outpatients rather than from the general population. Although individuals with HS or systemic inflammatory dermatoses were excluded from the control group, dermatology-based controls may differ from the general population in unmeasured lifestyle, metabolic, or healthcare-related characteristics, which may limit generalizability and contribute to residual confounding.

## Figures and Tables

**Table 1:** Demographic Characteristics of Patients with HS and Controls.

Variable	HS Patients (*n* = 176)	Controls (*n* = 132)	*p*-Value
Age (years), median (IQR)	32.0 (24.0–43.0)	31.0 (25.0–41.3)	0.662
Sex, Male/Female, *n* (% Male)	125/51 (71.0%)	94/38 (71.2%)	1.000
Height (cm), mean ± SD	171.9 ± 9.0	171.8 ± 8.2	0.878
Weight (kg), median (IQR)	87.0 (80.0–97.5)	78.0 (68.8–84.0)	**<0.001**
BMI (kg/m^2^), median (IQR)	29.4 (27.4–32.9)	26.1 (23.5–28.4)	**<0.001**

Mann–Whitney *U* test. Values are presented as median (interquartile range, IQR) or mean ± standard deviation (SD). The groups were comparable in age, sex, and height; weight and BMI were significantly higher in the HS group. Bold values indicate statistical significance (*p* < 0.05).

**Table 2:** Comparison of Serum Lipid Parameters and Atherogenic Indices Between the HS and Control Groups.

Variable	HS Patients (*n* = 176)	Controls (*n* = 132)	*p*-Value
Total cholesterol (mg/dL)	186.5 (159.3–207.2)	174.8 (153.0–202.6)	0.166
Triglycerides (mg/dL)	134.5 (92.3–184.5)	101.4 (69.9–152.9)	**<0.001**
HDL-cholesterol (mg/dL)	40.0 (35.4–46.2)	46.2 (39.4–55.5)	**<0.001**
LDL-cholesterol (mg/dL)	111.3 (93.7–129.4)	101.4 (83.4–118.7)	**0.034**
Non-HDL cholesterol (mg/dL)	143.0 (118.4–164.0)	123.2 (101.0–151.0)	**0.002**
AC	3.46 (2.70–4.31)	2.75 (1.94–3.56)	**<0.001**
CRI-I	4.46 (3.70–5.31)	3.75 (2.94–4.56)	**<0.001**
CRI-II	2.70 (2.12–3.27)	2.18 (1.55–2.84)	**<0.001**
AIP	0.16 (−0.04–0.35)	−0.01 (−0.20–0.19)	**<0.001**

Mann–Whitney *U* test. Abbreviations: HDL-C, high-density lipoprotein cholesterol; LDL-C, low-density lipoprotein cholesterol; NHC, non-high density lipoprotein cholesterol; AC, atherogenic coefficient; CRI-I and II, Castelli Risk Index I and II; AIP, atherogenic index of plasma. Bold values indicate statistical significance (*p* < 0.05).

**Table 3:** Comparison of lipid parameters and atherogenic indices between HS and control groups before and after adjustment for BMI.

Variable	Adj. Mean Patients *	Adj. Mean Controls *	Group Diff. (*β*, SE)	95% CI	t	*p* (BMI-Adj.)
Total cholesterol (mg/dL)	181.93	181.81	0.12 (4.86)	−9.44, 9.68	0.02	0.980
Triglycerides (mg/dL)	143.67	129.08	14.59 (9.20)	−3.52, 32.70	1.59	0.114
LDL-cholesterol (mg/dL)	111.88	107.41	4.48 (4.41)	−4.20, 13.15	1.01	0.311
Non-HDL cholesterol (mg/dL)	139.48	133.73	5.75 (4.87)	−3.83, 15.33	1.18	0.239
HDL-cholesterol (mg/dL)	42.45	48.08	−5.63 (1.52)	−8.62, −2.64	−3.70	**<0.001**
CRI-I	4.50	4.05	0.45 (0.17)	0.12, 0.79	2.67	**0.008**
CRI-II	2.78	2.44	0.35 (0.14)	0.08, 0.62	2.55	**0.011**
AC	3.50	3.05	0.45 (0.17)	0.12, 0.79	2.67	**0.008**
AIP	0.12	0.02	0.09 (0.04)	0.02, 0.17	2.58	**0.010**

Adjusted means, BMI-adjusted group differences (*β* coefficients), 95% confidence intervals, *t*-values, and *p*-values were obtained from multiple linear regression models * including study group and BMI as predictors. To account for multiple testing across the nine lipid parameters and lipid-derived indices, the Benjamini–Hochberg false discovery rate (FDR) procedure was additionally applied. In the unadjusted HS-versus-control comparisons, all associations that were statistically significant in the primary analyses remained significant after FDR correction. Following BMI adjustment, HDL-C (q = 0.002), CRI-I (q = 0.020), CRI-II (q = 0.020), AC (q = 0.020), and AIP (q = 0.020) remained statistically significant, whereas total cholesterol, triglycerides, LDL-C, and non-HDL-C remained non-significant. For descriptive assessment of between-group effect magnitude, the corresponding unadjusted Hedges’ g values were −0.60 for HDL-C, 0.51 for CRI-I, 0.46 for CRI-II, 0.51 for AC, and 0.54 for AIP. Because several of these indices share the same lipid components and CRI-I and AC are deterministically related (CRI-I = AC + 1), these findings represent overlapping rather than independent lipid signals. Bold values indicate statistical significance (*p* < 0.05).

**Table 4:** Lipid Levels and Atherogenic Indices According to Hurley Stage.

Variable	Stage I (*n* = 41)	Stage II (*n* = 63)	Stage III (*n* = 72)	Kruskal–Wallis *p*	Significant Pairwise Comp. *	Spearman ρ ^‡^	Trend *p*	FDR q ^§^
Total cholesterol (mg/dL)	179.7 (155.0–206.9)	183.0 (157.8–202.9)	198.0 (161.7–208.9)	0.180	—	0.134	0.077	0.098
Triglycerides (mg/dL)	118.5 (89.0–200.7)	117.8 (91.1–189.3)	142.0 (101.9–177.9)	0.655	—	0.061	0.422	0.422
HDL-cholesterol (mg/dL)	43.0 (38.6–50.0)	40.0 (34.4–46.2)	39.0 (35.4–45.2)	**0.033**	SI vs. SIII (*p* = 0.026)	**−0.174**	**0.021**	**0.031**
LDL-cholesterol (mg/dL)	104.0 (74.4–119.7)	110.0 (85.4–127.0)	114.0 (102.5–135.3)	**0.028**	SI vs. SIII (*p* = 0.036)	**0.201**	**0.007**	**0.017**
Non-HDL cholesterol (mg/dL)	139.0 (110.0–151.3)	137.6 (115.5–159.5)	150.6 (128.3–169.3)	0.045	—	**0.186**	**0.014**	**0.024**
CRI-I (TC/HDL)	4.01 (3.19–4.88)	4.40 (3.83–5.24)	4.86 (4.02–5.50)	**0.010**	SI vs. SIII (*p* = 0.009)	**0.226**	**0.003**	**0.008**
CRI-II (LDL/HDL)	2.35 (1.61–2.77)	2.64 (2.12–3.05)	3.00 (2.49–3.60)	**0.002**	SI vs. SIII (*p* = 0.003)	**0.262**	**<0.001**	**0.004**
Atherogenic coefficient (AC)	3.01 (2.19–3.88)	3.40 (2.83–4.24)	3.86 (3.02–4.50)	**0.010**	SI vs. SIII (*p* = 0.009)	**0.226**	**0.003**	**0.008**
AIP (log_10_[TG/HDL])	0.11 (−0.22–0.37)	0.12 (−0.07–0.32)	0.20 (0.08–0.35)	0.316	—	0.114	0.131	0.147
BMI (kg/m^2^)	29.2 (27.6–31.8)	30.1 (26.8–33.4)	29.4 (27.7–32.7)	0.997	—	−0.006	0.941	—

Abbreviations: SI, Stage I; SII; Stage II, SIII; Stage III. Values are presented as median (interquartile range, IQR). * Bonferroni-corrected *p*-value (3 comparisons); calculated only for variables with Kruskal–Wallis *p* < 0.05. ^‡^ Spearman rank correlation coefficient assessing the monotonic association with increasing Hurley stage (I–III). ^§^ Benjamini–Hochberg FDR-adjusted q-value across the nine lipid parameters and lipid-derived indices included in the ordinal trend analyses. Bold values indicate statistical significance (*p* < 0.05).

## Data Availability

The data used in this study were obtained from retrospective archive records. Due to privacy and ethical restrictions, the datasets are not publicly available but are available from the corresponding author upon reasonable request.
